# Anodal tDCS to Right Dorsolateral Prefrontal Cortex Facilitates Performance for Novice Jazz Improvisers but Hinders Experts

**DOI:** 10.3389/fnhum.2016.00579

**Published:** 2016-11-16

**Authors:** David S. Rosen, Brian Erickson, Youngmoo E. Kim, Daniel Mirman, Roy H. Hamilton, John Kounios

**Affiliations:** ^1^Creativity Research Laboratory, Department of Psychology, Drexel UniversityPhiladelphia, PA, USA; ^2^Music and Entertainment Technology Laboratory, Department of Electrical and Computer Engineering, Drexel UniversityPhiladelphia, PA, USA; ^3^Language and Cognitive Dynamics Laboratory, Department of Psychology, University of Alabama at BirminghamBirmingham, AL, USA; ^4^Laboratory for Cognition and Neural Stimulation, Perelman School of Medicine, University of PennsylvaniaPhiladelphia, PA, USA

**Keywords:** creativity, expertise, tDCS, jazz improvisation, dual-process model, neuroplasticity

## Abstract

Research on creative cognition reveals a fundamental disagreement about the nature of creative thought, specifically, whether it is primarily based on automatic, associative (Type-1) or executive, controlled (Type-2) processes. We hypothesized that Type-1 and Type-2 processes make differential contributions to creative production that depend on domain expertise. We tested this hypothesis with jazz pianists whose expertise was indexed by the number of public performances given. Previous fMRI studies of musical improvisation have reported that domain expertise is characterized by deactivation of the right-dorsolateral prefrontal cortex (r-DLPFC), a brain area associated with Type-2 executive processing. We used anodal, cathodal, and sham transcranial direct current stimulation (tDCS) applied over r-DLPFC with the reference electrode on the contralateral mastoid (1.5 mA for 15 min, except for sham) to modulate the quality of the pianists' performances while they improvised over chords with drum and bass accompaniment. Jazz experts rated each improvisation for creativity, esthetic appeal, and technical proficiency. There was no main effect of anodal or cathodal stimulation on ratings compared to sham; however, a significant interaction between anodal tDCS and expertise emerged such that stimulation benefitted musicians with less experience but hindered those with more experience. We interpret these results as evidence for a dual-process model of creativity in which novices and experts differentially engage Type-1 and Type-2 processes during creative production.

## Introduction

The study of improvisation is pertinent to any domain that requires adaptation, problem solving, and innovation. The ability to generate, execute, and evaluate choices in real-time can be seen in a range of scenarios from friends having a conversation, to surgeons operating in an emergency room, to musicians performing in a jazz club. In jazz, as in other domains, creative improvisation is developed through rigorous training and experience over many years. Research has begun to offer insights into the structural and functional neural changes that occur as this expertise is acquired (Beaty, 2015).

In the present study, we tested a dual-process model for understanding creativity (Rosen et al., in press) and jazz improvisation (Pressing, 1988; Johnson-Laird, 2002) by using transcranial direct current stimulation (tDCS) to modulate the quality of jazz pianists' improvisations. Based on our previous work (Rosen et al., in press), we hypothesized that musical improvisation involves a mixture of deliberate and unconscious processes and that the contributions of these two types of processes depend on the expertise of the performer. Our results show that tDCS can produce different effects on musical improvisation that depend on the performer's level of accumulated expertise, thereby supporting our dual-process model of creativity.

## Literature review

### Musical improvisation

Musical improvisation is sometimes cited as an ecologically valid creative task that does not benefit from increased cognitive control in contrast to standardized laboratory assessments of creativity (see Beaty, 2015 for a review). fMRI studies of musical improvisation suggest that widespread frontal-lobe deactivation, particularly in the right hemisphere, is characteristic of expert-level jazz musicians and that the magnitude of these deactivations is predicted by musicians' number of hours spent improvising (Pinho et al., 2014). Thus, expert jazz improvisation contradicts the view that creativity is primarily supported by top-down control, analytical processing (Nijstad et al., 2010; Baas et al., 2013), and executive function (Nusbaum and Silvia, 2011; De Dreu et al., 2012). Instead, neuroimaging studies of expert-level jazz improvisation suggest decreased activation of prefrontal and parietal cortices, increased activation of the default-mode network (posterior cingulate, medial prefrontal cortex, angular gyrus, etc.), and enhanced connectivity among prefrontal, premotor, motor, and default mode regions (Limb and Braun, 2008; Pinho et al., 2014, 2016). These activation and deactivation patterns are thought to represent a shift from top-down control to more automatic, bottom-up, implicit processing, which facilitates creative performance (Yang, 2015), not only for expert improvisers, but also in other creative domains and tasks (Jung et al., 2013; Chrysikou et al., 2014).

In a behavioral study of jazz improvisation, Rosen et al. (in press) reported that engaging more executive processing and cognitive control via explicit instructions to “be creative” significantly increased improvisation ratings for less experienced jazz musicians; however, more experienced jazz musicians did not show similar improvement. They interpreted these results as evidence for a dual-process model of creativity in which both unconscious, associative (Type-1) and deliberate, controlled (Type-2) processes can contribute to creative thought (e.g., Nijstad et al., 2010; Sowden et al., 2015) with the mixture of these types of processes determined by individual differences (e.g., expertise and personality) and context (e.g., instructions).

While improvising, one must manage rapid chord changes, note choices, appropriate rhythmic execution, and so forth. Type-2 processes activated by instructions (Green et al., 2015) facilitate performance for less experienced musicians by redirecting their attention to a goal of creative expression, recruiting strategies likely to yield a highly creative product and by avoiding the cognitive fixation (Howard-Jones, 2002) that can result from limited domain knowledge and proficiency. However, ramping up Type-2 processes does not improve creative performance for more experienced jazz musicians because experts rely more heavily on Type-1, implicit processes. Due to their extensive training and experience, experts develop enhanced domain-related functional connectivity (Pinho et al., 2014) reflecting a dominance of Type-1 processes or a near-optimal balance between Type-1 and Type-2 processes. Therefore, triggering additional Type-2 processing via creativity instructions does not significantly benefit experts' improvisations, as rated by expert judges (Rosen et al., in press).

### tDCS and creativity

tDCS is another cognitive modulation technique which may enhance creative performance. This technique applies a weak direct current to the scalp using two saline-soaked sponge electrodes. The electrical current is thought to alter neuronal membrane potentials, affecting the excitability of a targeted brain region (Zheng et al., 2011). It has been reported that anodal stimulation increases cortical excitability, and cathodal stimulation decreases cortical excitability (Nitsche and Paulus, 2001). In this study, we sought to extend findings of enhancement of cognitive (Coffman et al., 2014; Nelson et al., 2014) and creative production (Chrysikou et al., 2013; Mayseless and Shamay-Tsoory, 2015; Green et al., 2016) via tDCS to the domain of creative musical performance. It has been suggested that tDCS stimulation can differentially impact individuals depending on their baseline abilities and degree of expertise (Kadosh et al., 2010; Turkeltaub et al., 2012; Mayseless and Shamay-Tsoory, 2015). We therefore examined the effects of stimulation to right dorsolateral prefrontal cortex (rDLPFC) on the creativity of jazz improvisations in a sample of jazz pianists who had different levels of expertise.

Although several studies have examined the effects of tDCS on creativity and insight, the literature offers little clear evidence for its effectiveness as an enhancer of these abilities. Nevertheless, this small body of work has yielded some intriguing preliminary results. One of the earliest of these studies showed that participants were three times as likely to correctly solve an insight problem with concurrent bilateral stimulation to the anterior temporal lobes (ATL) when the cathode was over left ATL and the anode was over right ATL (Chi and Snyder, 2011). However, the stimulation was reliable only compared to sham—the effect was not significant when reversing the stimulation polarity (anode—left ATL, cathode—right ATL). Furthermore, the study did not determine whether participants' solutions really resulted from insight or whether they resulted from analytical thinking. (This was also a limitation of the study by Cerruti and Schlaug, 2009).

Other tDCS creativity research asked participants to generate a common or uncommon use for objects in pictures. Chrysikou et al. (2013) stimulated left or right inferior frontal gyrus (l-IFG, r-IFG) unilaterally with cathodal stimulation (the anode was placed on the contralateral mastoid) along with a sham condition. While not testing creativity directly, the authors reported that cognitive flexibility improved only with cathodal stimulation to the l-PFC in the uncommon uses condition such that reaction times and response omissions significantly decreased. Here, cathodal stimulation may have inhibited linguistic left-hemispheric dominance and induced hypofrontality of the l-PFC. This finding may be similar to those from neuroimaging studies of jazz improvisation that suggest that deactivation of PFC may benefit creative cognition by facilitating a flow state (Limb and Braun, 2008), characterized as feeling energized focus, complete engagement, and enjoyment in the process of the activity (Csikszentmihalyi, 1990).

Other studies have not been able to reproduce the beneficial effects of unilateral stimulation on creative tasks. Mayseless and Shamay-Tsoory (2015) found that bilateral stimulation with anodal tDCS over right inferior frontal gyrus (r-IFG) and cathodal tDCS over left inferior frontal gyrus (l-IFG) significantly increased flexibility and fluency in a verbal divergent thinking task. The opposite pattern of stimulation yielded no effect. Interestingly, in a second experiment, separately targeting l-IFG with cathodal stimulation or r-IFG with anodal stimulation did not impact divergent thinking scores. The authors hypothesized that the lack of an effect of unilateral cathodal stimulation to l-IFG, similar to Chrysikou et al. (2013), was potentially due to a difference in stimuli—pictures of objects may initially recruit more right-hemisphere brain areas (Corballis, 2003) while verbal stimuli initially engage a left-hemisphere network (Binder et al., 1997). It is also possible that the disparity in the measures of creativity make the comparison between these results problematic.

A recent study by Green et al. (2016) found that anodal high-definition tDCS administered to left frontopolar cortex, compared to sham, increased the likelihood of successfully validating analogy pairs whose words had a greater semantic distance. Here, the authors used semantic distance as a measure of creativity because a higher semantic distance indicates that the words are uncommonly paired, requiring participants to cast a broader search between terms to correctly identify their relationship. Thus, semantic distance may offer a glimpse into one type of verbal creativity, as it satisfies the common creativity definition “unusual and appropriate” (Sternberg, 1988). For the same stimulation paradigm in a verb-generation task, tDCS did not increase semantic distance; however, when combined with a cue to be creative, there was a significant interaction with tDCS increasing semantic distance of verb responses to a noun stimulus. With the creativity cue, there was evidence of increased activation of frontopolar cortex and other brain areas (Green et al., 2015). These researchers proposed that the neural intervention induced a creative state that enhanced participants' ability to generate semantically distant responses. However, the linguistic nature of the task promotes left-hemisphere dominance and may not generalize to other creative domains such as music.

### tDCS and music performance

Few studies have examined the effects of tDCS on creative performance. Even fewer have studied the impact of tDCS on creative performance in artistic domains. Though none of these investigations have examined musical creativity directly, two studies examined the effects of targeting motor cortex (C3 and C4) with tDCS on trained and untrained pianists' finger dexterity and fine motor control (Furuya et al., 2013, 2014). In the first study, concurrent bilateral tDCS to motor cortex improved keystrokes for untrained musicians but did not improve performance for professionals. Interestingly, some professional pianists who began training at a later age did show improvement for some movement features, indicating that the age at which pianists started training was positively correlated with the amount of finger-movement improvement from tDCS.

In the other study, Furuya et al. (2014) replicated these findings, displaying a ceiling effect on skilled musicians' improvement in fine motor control due to tDCS. As before, the untrained control participants demonstrated improvements in both the left and right hands when receiving concurrent bilateral brain stimulation to motor cortex in both conditions (anode—C3, cathode—C4; anode—C4, cathode—C3). Furthermore, placing the anode over the contralateral cortex and cathode over the ipsilateral cortex (relative to the hand one was performing with) degraded performance for professional pianists compared to the sham condition. These results provide further evidence for the expertise-dependent functional networks and organization, specifically within motor cortex. In contrast, for the control participants, either montage of bilateral stimulation to motor cortex improved motor control.

Evidently, tDCS can disrupt the optimized neural architecture of highly-trained musicians. Together, these studies suggest expert musicians' functional networks may be resistant to, or even hindered by, modulation by tDCS, especially anodal tDCS.

In the current experiment, we hypothesized that anodal stimulation of r-DLPFC during musical improvisation would enhance the performances of non-expert musicians while yielding neutral or negative effects for experts; however, we predicted that the inhibitory effects of cathodal tDCS would have the opposite effect, as deactivation of frontal cortex should disinhibit experts' optimized Type-1 performance networks.

## Methods

### Participants

Jazz pianists from local collegiate music departments, seminaries, bands, and jazz associations in the Philadelphia, PA region were recruited for this study. Due to the highly specialized nature of the sample population, we pursued subject recruitment for 6 months, stopping after we could find no more musicians who met our criteria for participation. Due to the within-subject design of this study, pianists were required to attend three experimental sessions each of which featured a different stimulation-type (anodal, cathodal, or sham). Of the 23 musicians recruited, 4 were not able to complete the study due to scheduling conflicts; 1 participant decided not to complete the study; 1 subject's data were not included due to an apparent, unreported, neurological problem. The remaining jazz pianists (*N* = 17) were free of neurological or psychiatric issues and were not taking any neurological or psychiatric medications. They also met the musical requirements: having improvised in a live jazz setting at least 3 times and having at least 10 years of musical training.

Four jazz experts were recruited to judge the improvisations after all of the experimental sessions were complete. These judges included a director of a collegiate jazz program, two jazz faculty members, and a professional jazz pianist and instructor. All jazz faculty worked at different universities in the Philadelphia area. All raters had more than 25 years of professional performance experience. Musicians and judges were given monetary compensation for their time.

### Experimental procedure

Participants were tested individually and completed the experiment in 3 sessions. Each session lasted approximately 1 h and was conducted at the Laboratory for Cognition and Neural Stimulation (LCNS) at The University of Pennsylvania in Philadelphia, PA. This study was approved by the University of Pennsylvania's Institutional Review Board. At the beginning of the first session, participants signed informed consent, 2 questionnaires as part of a separate study, a handedness inventory, and a mood survey. Upon completion of these surveys, participants were told that they were taking part in a study to examine the effects of tDCS on jazz improvisation without any mention of creativity or expertise. Each participant was given a brief overview of the tDCS equipment, electrodes, and setup while their heads were measured. Once the measurements were complete, participants were fitted with the tDCS electrodes.

**Figure 1:**
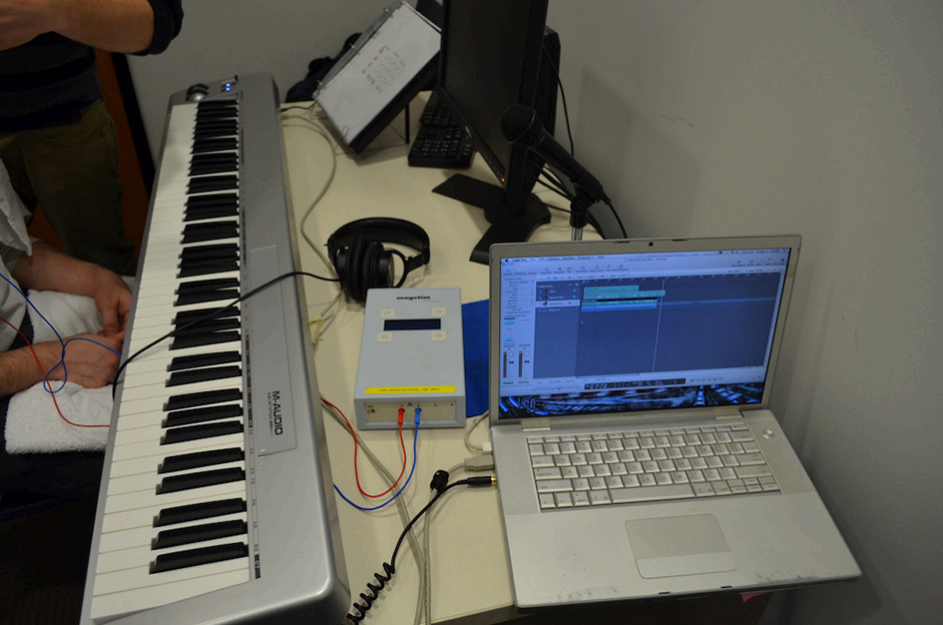
**Experimental setup**.

**Figure 2:**
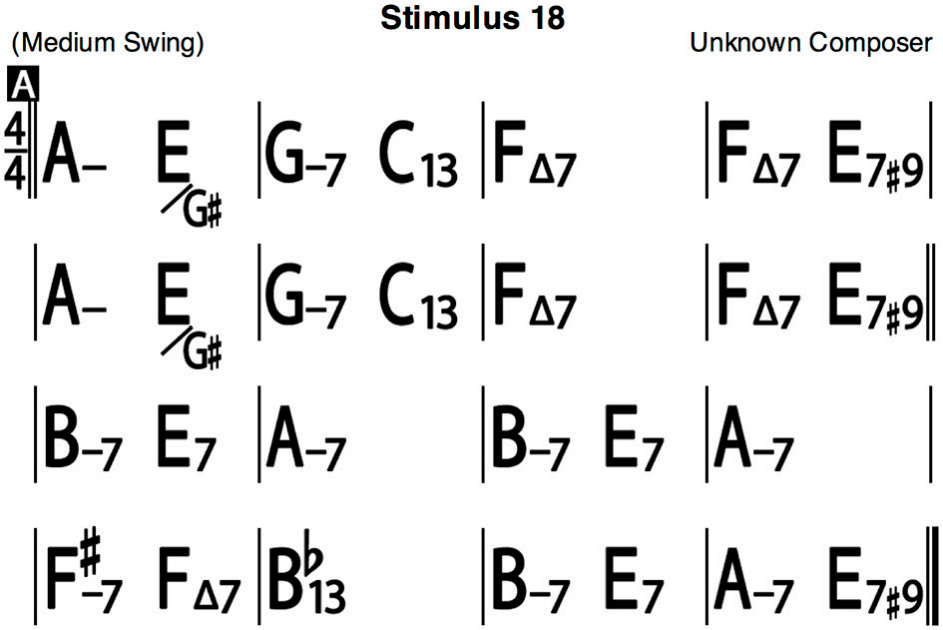
**Sample jazz lead sheet**.

At this point in each session, an M-Audio Keystation 88 USB MIDI Controller Keyboard (M-Audio, Cumberland, RI), sustain pedal, music stand, studio quality headphones, and a binder containing task instructions and jazz lead sheets (a visual representation of the chords of a song) were provided for the improvisation task. The experiment's improvisation and recording setup can be viewed in Figure 1. Instructions emphasized that pianists “should improvise as they would in a jazz setting.” Headphones were worn by the musicians for all improvisation “takes,” so that only the musician was able to hear the output of their improvisation, which was not audible in the room with the experimenter present. We did this to decrease the likelihood of self-consciousness among subjects that could occur if the researcher could hear the improvisations as they were performed. Musicians improvised over a 2-min “Dominant 7ths” exercise during inactive tDCS (electrodes worn but machine turned off) to ensure comfort in the recording environment and to allow for any volume adjustments between their piano and the backing tracks. Apple's *Logic Pro 9* v.9.1.8 (Cupertino, CA) music software recorded the improvisations, collected MIDI performance data, and provided musicians with a bass and drums audio accompaniment. Accompaniments were created through *iReal b* for Mac OS X v.2.8 (New York, NY), a practice tool with a full rhythm section for any properly formatted jazz chart (Figure 2).

Musicians were randomly assigned to one of six groups which determined stimulation order in their three sessions. The stimulation order was nearly counterbalanced, except for the sham/cathodal/anodal sequence which had one less subject. Musicians were instructed to sit quietly with eyes open while gazing at a fixation cross during the first 6 min of stimulation. The researcher presented the first lead sheet and reminded participants they were now going to improvise to 6, 16-bar jazz songs. Each song included 4 chord cycles and lasted approximately 2 min. The improvisation audio stimulus began with a 4-click count-in, and there were intervals of 15–20 s between stimuli. Musicians improvised with online tDCS for the first 4 takes and offline tDCS for the last 2 in each session. Only the final two offline takes from each session were rated and used in the subsequent analyses. Cognitive demands during online tDCS can influence offline, post-stimulation performance (Gill et al., 2015); therefore, the choice to only assess the final 2 takes was done in pursuit of maximal, task-specific, long-lasting tDCS effects.

**Figure 3:**
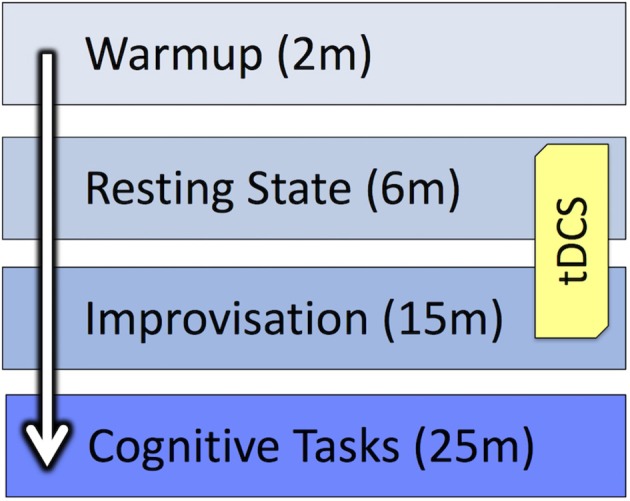
**Study design**.

After the improvisations were complete, the electrodes were removed, and musicians noted which performance they thought was their best. They were then presented with two creativity tasks: a Verb Generation Task (Prabhakaran et al., 2014) and a Compound Remote Associates test (Bowden et al., 2005) followed by a number Stroop test (Windes, 1968). Data from these tests have not yet been analyzed and are not included in the present report. Also, a post-tDCS survey was collected as part of another ongoing project to better understand how participants perceive the effects of brain stimulation (Kessler et al., 2012). A personality inventory and demographic survey, which included questions about participants' musical backgrounds, were administered during the final session. Figure 3 provides an overview of the study design.

Upon completion of data collection, the jazz improvisations (*n* = 102) recorded during sham (*n* = 34), anodal (*n* = 34), and cathodal (*n* = 34) stimulation were normalized to ensure the piano and accompaniment had the same relative volume levels across all takes (see Supplemental Materials for sample jazz improvisations). The order of the performances was pseudo-randomized for judging with the constraints that the same musician could not be heard consecutively or more than twice within a single judging block. Judging blocks began with an improvisation from an expert and novice improviser from 1 of the first 4 takes in the sham condition. These ratings were not included in the analysis. They were included as a reference point for raters, so they could familiarize themselves with the range of quality of the performances. To determine expert and novice clip selection, we split the participants into quartiles and randomly selected an improvisation from the top (200 or more live performances) and bottom (15 or less live performances) quartiles. Each judge rated the 102 improvisations and 10 baseline takes in 5 blocks of 22–23 improvisations each; however, only the final two, offline takes from each session were included in the analysis. Judging time for each block was approximately 45 min.

### Measures and instruments

Judges scored improvisations on creativity (CR), technical proficiency (TP), and esthetic appeal (AA) on a 7-point Likert scale according to the Consensual Assessment Technique (CAT) (Amabile, 1982). This technique has been used in hundreds of creativity studies and is based on the idea that evaluating a real product is not dependent on any single theory of creativity. Instead, this mode of assessment mirrors how creativity is determined in real-world domains (Baer, 2010). Critically, the CAT tasks experts in a domain to rate creative products relative to one another rather than against an absolute standard (e.g., a Miles Davis solo). This method has been used to assess the creativity of musical improvisation with high interrater reliability (De Dreu et al., 2012; Beaty et al., 2013).

The demographic musician questionnaire asked basic questions about participants' musical backgrounds and perceptions of the study improvisation task. This included: age; years of music and jazz training; primary performance genre (10 jazz, 2 rock, 2 classical, 1 folk/bluegrass, 1 electronica, 1 other); number of gigs; degree of comfort improvising jazz (*M* = 3.82, *sd* = 1.29); difficulty of the improvisation task (*M* = 2.41, *sd* = 1.18); ecological validity of the task (*M* = 2.41, *sd* = 0.94); individual practice routines; and experiences improvising in other genres. Values presented here are on a 5-point Likert scale. In the present report, we focus on age and the expertise variables.

### Transcranial direct current stimulation

A battery-powered constant DC stimulator (neuroConn DC-Stimulator Plus, neuroConn, Ilmenau, Germany) was used to deliver the stimulation current. Thin, saline-soaked sponges were used to interface the 5 × 5 cm rubber electrodes with the scalp. Electrode placement locations were determined using the International 10–20 System. The target-site electrode was placed on the F4 site, approximately overlaying r-DLPFC (Homan et al., 1987). The return electrode (Nasseri et al., 2015) was placed over the contralateral mastoid process. Though we targeted r-DLPFC, we acknowledge that other brain areas may have been directly or indirectly stimulated (Stagg et al., 2013). Additionally, changes in functional connectivity with tDCS have been shown with various imaging techniques including EEG, fMRI, and graph-theoretical approaches (Polanía et al., 2011a,b).

Unilateral monopolar stimulation ramped gradually to its final intensity of 1.5 mA over the course of 30 s. Stimulation began 360 s prior to the first improvisation to allow for stimulation to take effect prior to the experimental trials (Nitsche and Paulus, 2000). Stimulation continued for an additional 9 min while musicians continued to improvise (total time under stimulation = 15 min). Ramp-down to no stimulation was 30 s. In the sham stimulation condition, subjects received 30 s of stimulation before ramp-down. The anode/cathode placement was counterbalanced in the sham condition. Stimulation ended after the fourth trial in each session, and the final 2 trials were completed offline. Improvisation during stimulation was done to maximize tDCS effects on the offline performances (Gill et al., 2015), and stimulation length was decided based on previous reports that tDCS of 10 min or longer can have lasting effects for up to 1 h (Nitsche et al., 2008).

### Statistical analyses

We analyzed the impact of tDCS and expertise on jazz improvisation ratings using linear mixed-effects (LME) hierarchical regression models (Baayen et al., 2008) as implemented in the *lme4* software package (Bates et al., 2012) in R (Vienna, Austria). LME models simultaneously assess group-level and individual-level patterns within a single analysis, taking into consideration fixed (tDCS, expertise) and random-effect parameters. We included random intercepts for each subject and each stimulus (*n* = 6) to account for inter-individual variation and inter-item variation (Baayen et al., 2008; Mirman, 2016). Models included maximal random-effect structures that allowed the model to converge (Barr et al., 2013).

ANOVA model comparisons were used to determine the parameters that best predicted the improvisation ratings. That is, we first computed the model with only the intercept term followed by “session #” to test for practice effects across sessions. We then computed the model with each potential expertise parameter, age, music training, jazz training, and number of jazz gigs, keeping jazz gigs in the model as it was significantly predictive of improvisation ratings. Stimulation condition was included as an additional fixed effect and as an interaction term with expertise, testing our main hypothesis that tDCS would have differential effects based on expertise. Models were compared using the log-likelihood (LL) goodness-of-fit measure. Changes in −2LL are distributed as χ^2^ with degrees of freedom equal to the number of parameters added. For all model comparisons, the random effects structures were identical.

## Results

### Musician demographics and expertise analysis

The musicians were 19–34 years of age (*M* = 24.2, *sd* = 4.0), and participants were predominantly male (2 females). Expertise data was collected for years of music training (*M* = 17.17, *sd* = 4.26), years of jazz training (*M* = 7.29, *sd* = 4.57), and number of live jazz gigs performed (*M* = 108.53, *sd* = 125.26). The number of gigs covered a large range that spanned 2 orders of magnitude (3–400) and were skewed (skew = 1.95). The number of live jazz performances is an accurate descriptor of a musician's improvisational experience and expertise (Rosen et al., in press), and previous work has shown that the number of hours of improvisational experience is predictive of distinct brain-activation patterns beyond years of music training or age (Pinho et al., 2014). Because estimates of time spent improvising can be imprecise, we use the number of gigs as our expertise parameter, and we show that this measure significantly predicts pianists' improvisation ratings better than age, musical training, and even jazz training (see Table 1).

**Table 1:** **Chi-square difference tests for model comparisons**.

**Model parameters**	**Log-likelihood**	**Chi-squared (χ^2^)**	**Degrees of freedom (*df*)**	***P*-Value**
Baseline	−109.45	NA	NA	
Session #	−108.76	1.39	2	0.498
Age	−107.71	3.49	1	0.062
Music Training (years)	−108.13	2.65	1	0.104
Jazz Training (years)	−108.10	2.71	1	0.099
Expertise	−99.08	20.74	1	<0.001^***^
Expertise + tDCS	−98.74	0.68	2	0.713
Expertise x tDCS	−94.16	9.84	4	0.043^*^

Significance codes:

* p < 0.05,

*** p < 0.001.

Each model was tested against the Baseline model until Expertise significantly improved model fit. Then, the tDCS fixed-effect and interaction models were compared to Expertise. The best performing model included the interaction term, Expertise x tDCS, predicting quality scores significantly better than only Expertise.

We applied a natural logarithmic transformation to the number of jazz gigs. The power law of practice posits that skill increases logarithmically. Empirical evidence shows that improvement with practice is linear in a log-log space (Newell and Rosenbloom, 1981). For example, a musician's second performance gives them twice as much experience over the first, but the 401st performance is only a slight increase beyond the 400th. A secondary motivation for the logarithmic transformation was to improve model fit optimization for wide ranges of data with substantial skew (Zumel et al., 2014). Thus, when we reference “expertise parameter,” it is the natural logarithmic transformation of the number of live jazz gigs.

### Interrater reliability

The intraclass correlation coefficient (ICC) measured interrater reliability (IRR) for judges' ratings of CR, AA, and TP. Reliability was calculated such that values were computed for consistency where systematic differences between raters are considered to be irrelevant (McGraw and Wong, 1996). IRR was calculated for creativity (*ICC* = 0.81, *N* = 4), technical proficiency (*ICC* = 0.77, *N* = 4), and esthetic appeal (*ICC* = 0.84, *N* = 4). All scales had high reliability, as an *ICC* > 0.75 is excellent 0.40 to 0.74 is adequate to good, and <0.40 is poor (Fleiss, 1986).

### Scale-type correlations

The 3 scale types had highly significant positive correlations after averaging the four judges' ratings for each improvisation: CR and AA [*r*_(100)_ = 0.96, *p* < 0.01], CR and TP [*r*_(100)_ = 0.91, *p* < 0.01], AA and TP [*r*_(100)_ = 0.93, *p* < 0.01]. These high correlations between scale types may represent the interconnectedness of these three performance features, such that one is needed to express the others in a technically demanding domain like jazz improvisation. Thus, the individual CR, AA, and TP scale-type ratings were averaged to form a single “quality” rating for each improvisation. For further analyses and mixed-effect regression models, the quality rating composite score across judges and scales was the dependent measure for each improvisation.

### Descriptive statistics

Each musician performed 2 takes x 3 conditions (anodal, cathodal, sham); an overall quality rating was calculated for each take (*M* = 3.85, *sd* = 1.33). Quality ratings were approximately normally distributed (skew = 0.04), though displaying less peakedness and shorter tails due to negative kurtosis (kurtosis = −1.06). Scores ranged from 1.58 to 6.33, covering almost the entire range of the 7–point Likert scale. No single improvisation received the top score from all judges on all scales, indicating that scores were not clustered at the top or bottom end of the range, avoiding ceiling or floor effects. Although scale-type was not included in the LME regression analyses due to the extremely high correlations between scores on different scales, ratings on the CR scale (*M* = 4.04, *sd* = 1.34) were the highest, followed by TP (*M* = 4.00, *sd* = 1.28) and AA (*M* = 3.50, *sd* = 1.45).

### LME regression model comparisons

Table 1 displays the results of the model comparison difference tests. Variables thought to contribute to the model were tested against a baseline model. Session was the initial fixed effect, yielding no evidence of practice effects across sessions. For the domain-expertise parameters, age, years of music training, and years of jazz training failed to significantly predict improvisation quality. Expertise based on the number of live jazz performances, did significantly improve model fit. Keeping expertise in our model, we then tested the stimulation conditions' predictive abilities, which did not improve model fit beyond expertise (see Table 1). To test our hypothesis that tDCS would have differential effects for jazz musicians with varying levels of expertise, we tested the interaction between expertise and tDCS, which revealed a significant increase in the model fit. To estimate tDCS effects at the high end of the expertise scale, the same model was also refitted with Expertise rescaled so that the maximal number of gigs was 0 and fewer gigs were represented as negative numbers. The model below displays the parameters with the best fit after all comparisons (terms in parentheses are random effects):

$$\begin{array}{l} Quality\;Rating=\text{Expertise + tDCS + Expertise }\times \text{ tDCS}\\ \text{ + }\left(\text{1|Subject}\right)\text{ + (1|Stimulus}) \end{array}$$

### Fixed-effect parameters

**Figure 4:**
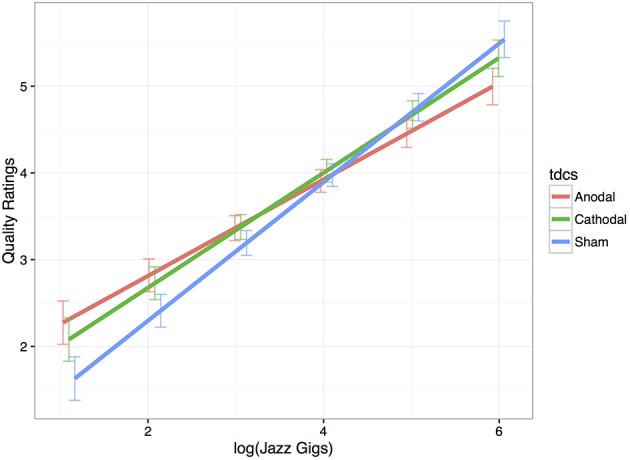
**Improvisation quality ratings as a function of Expertise × tDCS**. This model-based estimation displays musicians with less performance experience (left side of the x-axis) received higher ratings with anodal tDCS (red) compared to sham (blue). For the most experienced musicians, anodal stimulation decreased quality ratings compared to sham. Cathodal stimulation (green) did not significantly affect ratings. Error bars are displaying standard error.

As expected, expertise significantly increased improvisation ratings in the sham stimulation condition (*Estimate* = 0.80, *SE* = 0.11, *p* < 0.001). Thus, in the sham condition, there was an 0.80 increase in ratings per unit increase in expertise. Anodal and cathodal tDCS compared to sham did not affect the quality of performance for the sample as a whole (see Table 1); however, anodal tDCS significantly interacted with expertise-level. The significant negative interaction between tDCS and expertise reflects that quality ratings increase with anodal tDCS compared to sham for novices and significantly decrease for the experts (*Estimate* = −0.24, *SE* = 0.08, *p* = 0.002). Figure 4 displays this interaction. Furthermore, those musicians with the least experience benefited from anodal stimulation (*Estimate* = 0.91, *SE* = 0.32, *p* = 0.004), and those musicians with the most experience were hindered (*Estimate* = −0.54, *SE* = 0.20, *p* = 0.007). Here, ratings of our least experienced participants improved by almost a point when they had anodal stimulation, and the ratings of our most experienced participants decreased by about half a point when they had anodal stimulation compared to sham. The interaction between cathodal tDCS and expertise trended in the same direction but was not significant (*Estimate* = −0.14, *SE* = 0.08, *p* = 0.08). There was also a trend for cathodal stimulation to increase ratings for the least experienced musicians (*Estimate* = 0.60, *SE* = 0.32, *p* = 0.06), but experts' ratings were not affected (*Estimate* = −0.22, *SE* = 0.20, *p* = 0.28).

## Discussion

### Understanding the tDCS × expertise interaction

Neuroimaging studies of creativity and music improvisation report contradictory results with regard to the role of DLPFC. However, as new studies seek to tease apart this paradox, there is evidence that the DLPFC may have various functional roles dependent upon the creative task, goals, and individual-difference factors such as expertise (Pinho et al., 2014). It has been theorized that baseline abilities may differentially affect tDCS stimulation effects (Mayseless and Shamay-Tsoory, 2015), and that increased cognitive control is only advantageous in certain creative domains and situations (Chrysikou et al., 2014). In this study, we implemented a novel approach to examining the interaction between tDCS and jazz pianists' degree of domain-expertise with regard to the quality of their improvisations. We hypothesized that anodal tDCS to r-DLPFC would facilitate less-experienced musicians' performances, as novices display higher activity in frontoparietal executive systems (Pinho et al., 2014), relying on more explicit, conscious, Type-2 processes (Rosen et al., in press) compared to experts. In the cathodal stimulation condition, we predicted tDCS to amplify the benefits of hypofrontality to creativity (Chrysikou et al., 2013), jazz improvisation (Limb and Braun, 2008), and implicit, automatized, Type-1 processes acquired through expertise (Rosen et al., in press). Yet, we did not expect cathodal stimulation to improve novice performance because they rely more on top-down cognitive control and focused attention. Without engaging executive systems, less-experienced musicians would “presumably produce less adequate responses that are either too simplistic or esthetically inappropriate” (Pinho et al., 2016).

As an initial attempt to test these hypotheses about creative cognition in the ecologically valid domain of jazz improvisation, we applied unilateral tDCS to r-DLPFC as jazz pianists of various levels improvised to a series of chord changes across 3 sessions. As predicted, the musicians with the most professional experience received the highest improvisation ratings, consistent with data from past jazz improvisation studies (Beaty et al., 2013; Rosen et al., in press). These benefits of expertise align with theories of creative cognition in the performing arts in which musicians draw from a hierarchical structure of learned and novel ideas, form associative links between choices, and select and retrieve ideas activated in associative memory (Clarke, 1988). Thus, more experience develops finely-tuned, robust, functional neural networks.

There was no significant main effect of stimulation on the quality of jazz improvisations for the sample of jazz pianists; however, a highly-significant interaction between expertise and tDCS emerged in the anodal condition compared to sham, providing evidence for different modes of creative thought for experts and novices. Anodal tDCS improved performance for the least-experienced musicians relative to sham stimulation, and the opposite effect was obtained for the most-experienced musicians such that their performance was hindered relative to the sham condition.

These results suggest that anodal stimulation may increase the efficacy of r-DLPFC processes that are recruited during improvisation, allowing explicit top-down control and action selection (Nijstad et al., 2010) when novices' associative processes, knowledge structures and memory systems are insufficient for high-level, automatized performance (Pinho et al., 2016). De Dreu et al. (2012) reported that working memory (WM) in cellists predicted improvisation ratings over time, such that higher WM led to increased scores on subsequent takes. Thus, one explanation for these findings is that Type-2, executive processes that are critical to domain-general creativity such as working memory (Fregni et al., 2005; Boggio et al., 2006), attention (Coffman et al., 2014), inhibitory control (Javadi and Walsh, 2012), and visuospatial memory (Jeon and Han, 2012) are improved when anodal tDCS targets r-DLPFC. Still, the most recent meta-reviews do not provide evidence for benefits to working memory in healthy adults with anodal tDCS targeting r-DLPFC (Mancuso et al., 2016). It should be noted that the plethora of cognitive functions associated with DLPFC cannot be individually targeted with tDCS; therefore, we cannot ascertain how each executive process contributes to the modulation of novice jazz improvisation performance without combining tDCS with other techniques.

Another possibility is that the network of distant brain areas that are functionally connected to the stimulation area during improvisation are also affected by tDCS (Polanía et al., 2011a; Stagg et al., 2013). These downstream effects are likely to amplify the functional connectivity (Green et al., 2015) between prefrontal, premotor, and motor areas, potentially strengthening these networks to a point where they appear similar to more-experienced musicians. However, using this logic, we should have seen comparable improvement among experts. Furthermore, anodal tDCS may synchronize several brain regions that comprise a functional network if they are connected to the stimulation site (Kunze et al., 2016). This has been displayed through increased theta coherence between frontal and parietal lobes (Polanía et al., 2011a; Notturno et al., 2014). Interestingly, Gruzelier (2014) reports that neurofeedback training aimed to increase theta coherence, benefits musicians' creative performance such that training was associated with improvement in 9 of 13 performance criteria including interpretative imagination, expressive range, stylistic accuracy, technical security, rhythmic accuracy, tonal quality, and spectrum, deportment, emotional commitment and conviction, and the ability to cope with situational stress. It is thought that the role of theta coherence integrates widely distributed neural networks that underlie creativity (Gruzelier, 2009). This is another possible mechanism underlying the increases in improvisation scores for less-experienced musicians with anodal tDCS.

Based on the literature, we did not expect prefrontal anodal stimulation to assist the experts because the executive processes that they instigate are no more effective than, and may be inferior to, experts' typical emphasis on Type-1 processes associated with frontal-lobe deactivation (Limb and Braun, 2008; Liu et al., 2012; Pinho et al., 2014). Once enough domain expertise is gained, disinhibition and decreased cognitive control is an effective approach toward improvisation proficiency (Pinho et al., 2016). Thus, the anodal stimulation disrupted the trained neural networks of the most-experienced musicians. tDCS may have facilitated the recruitment of explicit processes that are normally inhibited, similar to what happens when one attends to the components of a well-learned skill, causing performance decrements (Beilock et al., 2002) and “choking” (Gray, 2004).

The interaction between expertise and cathodal tDCS was not significant, though there was a trend in the same direction as in the anodal stimulation condition, facilitating novice and hindering expert performance. Furthermore, we expected any impact of cathodal stimulation to have reverse effects of anodal stimulation with beneficial effects for the more-experienced jazz musicians, amplifying deactivations of prefrontal cortices that occurs as one gains expertise. There are a few reasons why we may not have seen the expected effect. First, cathodal stimulation does not reliably produce inhibitory behavioral effects (Jacobson et al., 2012). Compensation from other brain areas within functional networks may occur in some cognitive domains, masking the inhibitory behavioral effects of applying the cathode to one node of a network. We posit that improvisation performance gains via increased activation of compensatory networks are expertise-dependent. This would explain the trend for increases in quality ratings for less-experienced musicians but not experts. We propose a very different mechanism underlying the facilitation of performance with cathodal stimulation compared to similar improvements with anodal stimulation for novices. While anodal stimulation increased the efficacy of DLPFC's executive processes which novices routinely engage, we hypothesize that cathodal stimulation caused less experienced musicians to lessen their prefrontal dominance and cognitive control and recruit other brain areas within their functional networks (dorsal premotor cortex, medial prefrontal cortex, supplementary motor area), more so than normal. Thus, it is possible that cathodal stimulation allows novices to perform using a more bottom-up approach through downstream activations of this compensatory network. With regard to experts, past studies have shown that during improvisation musicians with more experience show greater deactivations of DLPFC (Pinho et al., 2014). Although we had hypothesized that cathodal tDCS would amplify these effects, it appears that cathodal stimulation does not further downregulate executive systems in such a way that would alter the optimal functional networks engaged by expert musicians.

Lastly, we did not find that cathodal stimulation facilitated expert-level jazz improvisatory performance. Of course, the question regarding the inhibitory effects of cathodal stimulation is a relevant one here, as well. Although motor studies consistently see inhibition of brain areas beneath the cathode, such evidence is rare for non-motoric cognitive studies. As mentioned, Jacobson et al. (2012) theorized that the lack of cognitive inhibition may reflect the complexity of cognition in that other brain areas in a rich neural network may serve as a buffer against potential disruption. Beyond that, one possibility is that expertise produces robust functional networks that are resistant to change from modulation techniques such as tDCS or explicit instructions (Rosen et al., in press). However, anodal stimulation did significantly impair expert performance. Unfortunately, with only 17 musicians, we were not able to determine whether the differences between stimulation conditions at each expertise-level were significant. Still, we report significant differential effects of tDCS on the quality of jazz improvisations for musicians with the highest and lowest degrees of expertise.

Another possible explanation for the lack of a significant positive impact of cathodal tDCS on seasoned jazz musicians may be analogous to studies examining pianists' finger dexterity and motor control (Furuya et al., 2013, 2014). In these studies, only untrained control participants and players that commenced training at an older age saw gains in finger dexterity with stimulation to motor cortex. These results “indicate robustness of the motor system of pianists against the tDCS intervention, being likely to reflect an early optimization of neuroplasticity” (Furuya et al., 2013). This would be a case in which previous experience results in an optimized system that imposes a ceiling effect that tDCS cannot improve upon. In the present study, this optimized system would consist of Type-1 improvisation mechanisms that develop over decades of jazz improvisation. If, for experts, deactivation of r-DLPFC is a critical component of this network, it follows that cathodal stimulation would not further inhibit this region in a way that would enhance expert performance.

While we do not present these results as the definitive evidence of the impact of tDCS on jazz improvisation and musical creativity, they are important for understanding the processes engaged by novices and experts in pursuit of creativity in real-world domains. To date, brain stimulation studies of creativity have relied too heavily on standardized assessments such as the Alternate Uses Test or the Remote Associates Test. The development of practical methods for enhancing creativity depends on further research in ecologically valid studies, for example, math (Kadosh et al., 2010; Hauser et al., 2013), reading (Turkeltaub et al., 2012), and music and the arts. In particular, such work could have powerful implications for music education and the enhancement of musical creativity as instructors can leverage knowledge about music cognition in their training programs and curricula.

### Limitations

This study has some limitations that future research will need to address. First, the neurological and psychological requirements of tDCS participants, multiple test sessions, and the highly specialized population led to our relatively small sample size: only 17 jazz pianists completed all three sessions. Nevertheless, each musician contributed 6 improvisations rated by expert judges, two with cathodal, anodal, and sham stimulation, for a total of 102 rated improvisations. In spite of the relatively small sample, the interaction effect between tDCS and expertise still led to highly significant results partly due to the within-subject stimulation design. Still, it is important to note that this is the first evidence of tDCS influencing the quality of music performance, and this effect requires replication, especially because lack of power (due to small samples) can lead to over-estimation of effect sizes (Button et al., 2013).

In addition, our sample of jazz pianists included only a moderate range of age and expertise because recruitment was limited by age (older adults producing different responses to tDCS; Fujiyama et al., 2014). Although jazz musicians ranged from college undergraduates with under 10 gigs to professional adults with 400 gigs, the experiment did not include the most seasoned jazz professionals who have been performing over the course of decades—the masters. Therefore, it is unclear how the present results may extrapolate to the most experienced musicians, though there is no evidence that the inclusion of such experts would have altered the results.

In this experiment, as in many tDCS studies, localization of the tDCS current is a concern as the pattern of current flow can influence various cortical regions contingent upon individual differences in the geometry of the sulci and gyri (De Berker et al., 2013) and characteristics of soft tissue and bone mass (Datta et al., 2009). However, in the present study, even if the electrode montage we employed stimulated additional or other brain areas that were not considered, our central finding that brain stimulation differentially affected the performances of musicians with greater and lesser experience still holds. The basic implications for a dual-process model of creativity would still apply. In addition, one could question our decision to only include the offline improvisations in the analysis. The decision to exclude the online takes was done a priori based on work by Gill et al. (2015) which reports that effects of offline tDCS are enhanced when the online and offline tasks require the same cognitive processes. When tDCS began, there was a resting-state period lasting 6 min. During this time, it is impossible to determine what kind of processes, thoughts, or mental-states had been occurring, altering the initial impact of tDCS. Thus, in this experiment, we wanted to give musicians plenty of time to engage the cognitive processes used during improvisation with tDCS, in hopes of maximizing the effects of stimulation offline and on the subsequent cognitive tasks. To date, there are no reports comparing the effects of tDCS for online and offline performance. We plan to examine these differences and the time-course of the effects of stimulation in future research.

Although we examined performance in the real-world musical domain of jazz improvisation, the ecological validity of this study may have been somewhat lessened by the stimuli and setting. The chord sequences were loosely based on 16-measure segments of jazz standards, often shifting keys to make them more novel to the performers. While a melody is typically provided on a jazz lead sheet, we did not include a written melody, or “head,” as we did not want sight-reading skills to interfere with one's ability to improvise. While attempting to limit confounding variables, it is unknown how the omission of melodies may have altered the underlying improvisation processes. Additionally, the computer-generated accompaniment did not respond to musicians and had a static tempo; therefore, the soloist could not have the interactions that they would have had in a live jazz setting (Monson, 2009).

## Conclusions

The present study is a first attempt to explore the effects of tDCS on jazz improvisation, a demanding, ecologically valid form of creative expression. Here, we report that brain stimulation differentially influences the ratings of musicians' improvisations dependent upon their degree of expertise. Anodal stimulation to r-DLPFC significantly increased performance quality for the less-experienced pianists while hindering it for those with the most experience. These results provide evidence supporting a dual-process creativity model in which the recruitment of Type-1 and Type-2 processes differs for experts and non-experts. This provides an insight into the neuroplasticity associated with expertise in musical improvisation which may extend to other domains, both artistic and non-artistic.

## Author contributions

DR, BE, YK, RH, and JK contributed to the conception and design of this work. DR and BE, collected all data. Data analysis and interpretation was conducted by DR, BE, DM, RH, and JK. DR drafted the article, and DM, RH, YK and JK provided critical revisions of the article. Final approval of the version to be published was given by DR, BE, YK, DM, RH, and JK.

## Funding

This project was funded by Drexel University's ExCITe Center - Seed Project 2013-2253.

### Conflict of interest statement

The authors declare that the research was conducted in the absence of any commercial or financial relationships that could be construed as a potential conflict of interest.
