# Supplementary material for: Anodal tDCS to Right Dorsolateral Prefrontal Cortex Facilitates Performance for Novice Jazz Improvisers but Hinders Experts
Source: Front Hum Neurosci. 2016 Nov 16;10:579. doi: 10.3389/fnhum.2016.00579 (PMC5110534; doi:10.3389/fnhum.2016.00579)
Supplement: Supplementary file 1 [file DataSheet1.DOCX]

Supplementary Material

Anodal tDCS to right dorsolateral prefrontal cortex facilitates performance for novice jazz improvisers but hinders experts

David S. Rosen^1*^, Brian Erickson^1^, Youngmoo E. Kim^2^, Daniel Mirman^3^, Roy H. Hamilton^4^, John Kounios^1^

^1^Creativity Research Laboratory, Drexel University, Department of Psychology, Philadelphia, PA, USA.

^2^ Music and Entertainment Technology Laboratory, Drexel University, Department of Electrical and Computer Engineering, Philadelphia, PA, USA.

^3^Language and Cognitive Dynamics Laboratory, University of Alabama at Birmingham, Department of Psychology, Birmingham, AL, USA.

^4^Laboratory for Cognition and Neural Stimulation, University of Pennsylvania, Perelman School of Medicine, Philadelphia, PA, USA.

*** Correspondence:**David S. Rosen
Drosen@drexel.edu

# Supplementary Audio

There are 6 jazz piano improvisations included in the supplementary materials. Audio clips 1-3 are example of highly rated improvisations. Audio 1 = Anodal, Audio 2= Cathodal, and Audio 3 = sham.

Audio clips 4-6 were examples of poorly rated performances. Audio 4 = Anodal, Audio 5 = Cathodal, Audio 6 = sham.
